# Nitrous‐oxide‐induced polyneuropathy and subacute combined degeneration of the spine: clinical and diagnostic characteristics in 70 patients, with focus on electrodiagnostic studies

**DOI:** 10.1111/ene.16076

**Published:** 2023-09-27

**Authors:** L. T. Hassing, F. Y. Jiang, R. Zutt, S. Arends

**Affiliations:** ^1^ HagaZiekenhuis, Department of Neurology The Hague The Netherlands; ^2^ HagaZiekenhuis, Department of Radiology The Hague The Netherlands

**Keywords:** electrodiagnostic studies, nitrous oxide, polyneuropathy, subacute combined degeneration of the spine

## Abstract

**Background and purpose:**

Nitrous oxide (N_2_O) induced neurological symptoms are increasingly encountered. Our aim is to provide clinical and diagnostic characteristics with a focus on electrodiagnostic studies.

**Methods:**

Patients with neurological sequelae due to N_2_O presenting in our hospital between November 2018 and December 2021 reporting clinical and diagnostic data were retrospectively reviewed.

**Results:**

Seventy patients (median 22 years) were included. Median N_2_O usage was 4 kg/week during 12 months. Patients’ history revealed a higher rate of sensory symptoms compared to motor (97% vs. 57%) and 77% walking difficulties. Clinical diagnosis was polyneuropathy (PNP) in 44%, subacute combined degeneration (SCD) of the spine in 19%, both in 37%. Median vitamin B12 level was low (159 pmol/L), normal in 16%. The median methylmalonic acid was increased (2.66 μmol/L). Electrodiagnostic abnormalities were observed in 91%, with 72% fulfilling axonal PNP criteria, 20% showing mild to intermediate slowing. One patient fulfilled demyelinating PNP criteria not related to N_2_O abuse (Charcot−Marie−Tooth type 1a). More prominent motor nerve conduction abnormalities were found; lower limbs were more affected. In 64% with normal conduction, myography showed signs of axonal loss. Magnetic resonance imaging showed cervical myelopathy in 58% involving generally five to six segments.

**Conclusions:**

Nitrous oxide (N_2_O) leads to neurological symptoms by causing PNP and/or SCD primarily involving the legs. Distinguishing PNP and SCD clinically was shown to be insufficient. Electrodiagnostic studies showed axonal PNP. Demyelinating PNP due to N_2_O abuse was not present in our cohort. Therefore, further diagnostic work‐up is warranted if demyelinating features are present.

## INTRODUCTION

Nitrous oxide (N_2_O) is increasingly used as a recreational drug worldwide [1, 2, 3, 4, 5, 6]. In 2021 in the Netherlands 7.9% of the general population >18 years had used N_2_O, and recreational N_2_O use was even higher amongst students (lifetime prevalence 28.3%; last year prevalence 8.9%) [7].

Usage of N_2_O can cause functional vitamin B12 (VB12) deficiency resulting in severe neurological disorders of both the central and peripheral nervous system [8, 9]. Clinical manifestations are subacute combined degeneration (SCD) of the spinal cord, polyneuropathy (PNP) and neuropsychiatric symptoms [8, 10]. If N_2_O is stopped promptly and VB12 deficiency is treated adequately, symptoms can be reversible [6, 11, 12]. Unfortunately, many patients suffer from residual symptoms resulting in significant disability with a large impact on this relatively young and growing population [12, 13, 14, 15]. Literature on N_2_O abuse primarily focuses on N_2_O‐induced SCD. Recently more has been published about N_2_O‐induced PNP consisting of primarily axonal PNP, combined axonal and demyelinating PNP and rarely a pure demyelinating PNP [5, 6, 12, 16, 17, 18, 19, 20, 21, 22, 23, 24, 25].

Still, little is known about the clinical characteristics of N_2_O‐induced PNP and/or SCD and the correlation between clinical presentation and outcome of diagnostic examinations. In addition, only few data are published about electrophysiological features of N_2_O‐induced PNP. Therefore, the aim of this study was to evaluate these characteristics in a large cohort of patients with special focus on electrodiagnostic studies including myography.

## METHODS

A retrospective analysis was performed of patients (≥18 years) who visited our large urban teaching hospital between November 2018 and December 2021 diagnosed with N_2_O‐induced PNP and/or SCD, based on clinical presentation in combination with low functional VB12 levels and/or the patients acknowledged using N_2_O and by exclusion of other causes. Patients were identified with the use of our electronic patient database from the Department of Neurology at the HagaZiekenhuis, The Netherlands.

Clinical data, including demographics and detailed information on the amount (average number of 2 kilograms N_2_O tanks/week, with one balloon estimated to contain 8 gram N_2_O) and frequency of N_2_O use, were collected. Standardized laboratory testing included VB12 and methylmalonic acid (MMA). Levels of VB12 were divided into normal (>250 pmol/L), low end of normal (160–250 pmol/L) and low (<160 pmol/L). In the case of symptoms suggesting central nerve damage a magnetic resonance imaging (MRI) of the (cervical) spine was performed according to our local protocol (including sagittal T1/T2 and short tau inversion recovery together with an axial T2 weighted sequence). All MRI scans were reviewed by a neuroradiologist.

Electrodiagnosis (EDx) was performed according to the local EDx protocol. This protocol consisted of motor nerve conduction (median/abductor pollicis brevis muscle, peroneal/extensor digitorum brevis muscle and tibial/abductor hallucis muscle, including F waves) and antidromic sensory nerve conduction (median/index or middle finger, ulnar/little finger, superficial peroneal and sural nerve), Hoffmann reflex (tibial/soleus muscle) and myography (tibialis anterior muscle and/or interosseous dorsalis pedis muscle). The limbs of patients were not actively warmed up and values were not corrected for temperature. All electrophysiological measurements were compared to locally used nerve conduction study (NCS) reference values [26]. Axonal PNP was defined as reduced sensory and/or motor amplitudes (<80% of lower limit of normal) in at least two nerves, without demyelinating features [19, 26]. Myography was not part of the criteria for axonal PNP. PNP was considered demyelinating if demyelinating features were present in at least two nerves, as specified by the European Academy of Neurology and Peripheral Nerve Society (EAN/PNS) chronic inflammatory demyelinating polyneuropathy electrodiagnostic criteria [27]. These criteria can be found in Data S1.

Patients were clinically classified in one of the following categories, based on history taking and neurological examination: (i) PNP, (ii) SCD of the spinal cord and (iii) combination of PNP and SCD. Clinical PNP was defined according to the American Association of Electrodiagnostic Medicine definition for clinical research [28], with neuropathic symptoms (sensory and/or motor) together with normal to absent reflexes and/or decreased distal sensation and/or distal muscle weakness. Patients were concluded to have clinical SCD if at least one of the following signs was present: abnormal gnostic sensibility with normal vital sensibility, abnormal Romberg's sign, sensory level, positive Lhermitte sign, hyperreflexia, Babinski sign and/or spasticity [29]. In the case of clinical features of both, this was concluded to be a combination of PNP and SCD.

All data on demographic and clinical presentation, laboratory tests, MRI data and EDx findings are presented as median and range. All analyses were performed using SPSS (version 24, Armonk, NY, USA; IBM Corp.). Intergroup comparisons were made with the one‐way ANOVA test in the case of a numerical variable and the chi‐squared test in the case of a categorical variable. *p* values of less than 0.05 were considered to be statistically significant. The study protocol was approved by the regional ethics committee (METC ZuidHolland, number G20.112).

## RESULTS

### Demographics

#### Demographic baseline

In total 70 patients were included, 45 male (64%), 55 non‐Caucasian (79%). The median age was 22 years (range 18–50). The average use of N_2_O was 4.0 kg/per week (range 0.1–56), with a median usage duration of 12 months (range 1–96). The median onset of symptoms was 35 days (range 1–552) before presentation at the hospital (PNP group 52.5 days; SCD group 15 days). All of these variables were not statistically different between groups. Admission at the ward of the hospital was necessary in 20 patients (30%) due to the severity of symptoms (Table 1). Twenty‐eight patients (40%) were referred to a rehabilitation doctor.

**TABLE 1:** Demographics.

	Clinical diagnosis			
	Isolated PNP, *n* = 31	Isolated SCD, *n* = 13	Combined PNP and SCD, *n* = 26	Total, *n* = 70
Age in years, median (range)	22 (18–42)	24 (18–35)	22 (19–50)	22 (18–50)
Gender (male/female)	23 (74%)/8 (26%)	7 (54%)/6 (46%)	15 (58%)/11 (42%)	45 (64%)/25 (36%)
Background (Caucasian/non‐Caucasian)	8 (26%)/23 (74%)	3 (23%)/10 (77%)	4 (15%)/22 (85%)	14 (21%)/53 (79%)
N_2_O use in kg/week, median (range)	3.0 (0.10–14)	3.0 (0.1–38)	5.6 (0.1–56)	4.0 (0.1–56)
Total N_2_O exposure time in months, median (range)	12 (1–72)	7 (1–84)	18 (4–96)	12.0 (1–96)
Intoxication				
Alcohol	14/28 (50%)	8/13 (62%)	14/24 (58%)	36/65 (55%)
Smoking	18/27 (67%)	7/13 (54%)	14/24 (58%)	39/64 (61%)
Drugs	7/27 (26%)	3/12 (25%)	7/24 (29%)	17/63 (27%)
Cocaine	1	3	3	7
Cannabis	4	1	4	10
XTC/MDMA	3	1	0	5

Abbreviations: PNP, polyneuropathy; SCD, subacute combined degeneration; XTC/MDMA, ecstasy/3,4‐methylenedioxymethamphetamine.

#### Follow‐up

All patients were advised to stop inhaling N_2_O and were started on either intramuscular injection or oral hydroxocobalamin according to local protocol. The median follow‐up was 12 weeks (range 0–56 weeks). Eighteen patients (26%) did not show up at their follow‐up appointment at around 3 months. Of the remaining 52 patients (74% of the total cohort) with at least a follow‐up at 3 months 16 (31%) reported full recovery, and 26 (50%) reported only mild symptoms (mild paresthesia or hypoesthesia not interfering with daily activity). Ten patients (19%) still experienced invalidating symptoms: nine of them experienced foot drop with difficulty walking and one patient was unable to work due to cognitive problems related to N_2_O abuse. In total 28 patients (40%) were lost to follow‐up since another 10 patients (14%) were lost to follow‐up after the appointment at 3 months (either at the neurologist or rehabilitation physician).

### Clinical presentation

#### Clinically isolated PNP

Thirty‐one patients (31 patients) were clinically diagnosed with isolated PNP. According to our clinical definition of PNP all patients reported sensory complaints of which the majority were in both legs and arms (77%). Four patients (13%) had a positive Romberg sign of which one had a myelopathy with normal EDx and two had a normal MRI but abnormal EDx. Weakness was reported in 14 patients (45%) with objective weakness in eight (26%) on neurological examination (median Medical Research Council sum score of 60, range 42–60). Seventeen patients (55%) reported isolated sensory symptoms. Hyporeflexia was seen in 22 patients (71%). Twenty patients (65%) reported difficulty walking, of whom one patient was found on the ground with impaired consciousness and inability to stand up. Four patients (13%) experienced difficulties with micturition (inability to completely empty the bladder) of whom all had a normal MRI of the cervical spine with an abnormal EDx. Three (10%) suffered obstipation.

#### Clinically isolated SCD of the spinal cord

Thirteen patients (19%) were clinically diagnosed as SCD. According to our clinical definition of SCD, six patients (46%) had pathological reflexes (either Babinski sign and/or hyperreflexia), six patients (46%) had a sensory level (all with a thoracic level between the third and twelfth thoracic), seven patients (54%) had severe deficit in proprioception and three patients (23%) reported Lhermitte sign. Eight patients (62%) showed a combination of these signs. Seven patients (54%) had a positive Romberg.

#### Combined PNP and SCD of the spinal cord

A combination of PNP and SCD was clinically diagnosed in 26 patients (37%). The majority of reported symptoms were sensory signs (25 patients, 96%), walking difficulties (22 patients, 85%) and muscle weakness (17 patients, 66%, all with objective muscle weakness with a median Medical Research Council sum score of 58 [range 48–60]). Neurological examination showed sensory deficits, both gnostic (21 patients, 81%) and vital (23 patients, 88%), a sensory thoracic level (seven patients, 27%) and positive Romberg's sign (17 patients, 65%), hyporeflexia of the lower limbs (25 patients, 96%) and Babinski sign (four patients, 15%). The majority of patients had a combination of hyporeflexia and severely disturbed propriocepsis (16 patients, 62%). Seven patients (27%) reported micturition problems; detailed information on the type of micturition problems was lacking. MRI of the cervical spine was performed in all and four patients (57%) showed a myelopathy.

See Table 2 for a detailed overview of all clinical characteristics.

**TABLE 2:** EDx, MRI data, clinical data and laboratory findings.

	Clinical diagnosis			
	Isolated PNP, *n* = 31	Isolated SCD, *n* = 13	Combined PNP and SCD, *n* = 26	Total, *n* = 70
Medical history				
Sensory symptoms only LL	7 (23%)	1 (8%)	6 (23%)	14 (20%)
LL + UL	24 (77%)	11 (85%)	19 (73%)	54 (77%)
Muscle weakness only LL	4 (13%)	6 (46%)	9 (35%)	19 (27%)
LL + UL	10 (32%)	3 (23%)	8 (31%)	21 (30%)
Difficulty walking	20 (65%)	12 (92%)	22 (85%)	54 (77%)
Micturition problems	4 (13%)	3 (23%)	7 (27%)	14 (20%)
Defecatory problems	3 (10%)	2 (15%)	2 (8%)	7 (10%)
Cognitive deficits	4 (13%)	2 (15%)	3 (12%)	9 (13%)
Days with symptoms before first presentation at the hospital, median (range)	52.5 (1–552)	14 (7–120)	30.5 (1–552)	33 (1–552)
Neurological examination				
MRC‐SS median (range)	60 (42–60)	58 (52–60)	58 (48–60)	60 (42–60)
Sensory deficits				
Decreased vital sensibility only LL	14 (45%)	5 (39%)	11 (42%)	30 (43%)
LL + UL	15 (48%)	6 (46%)	12 (46%)	33 (47%)
Decreased propriocepsis only LL	0	6 (46%)	3 (12%)	19 (27%)
LL + UL	0	1 (8%)	4 (15%)	4 (6%)
Reduced/absent vibration sense only LL	7 (23%)	9 (69%)	14 (54%)	32 (46%)
LL + UL	5 (16%)	1 (8%)	3 (12%)	9 (13%)
Sensory level^a^	0	6 (46%)	7 (27%)	13 (19%)
Romberg's sign	4 (13%)	7 (54%)	17 (65%)	28 (45%)
Lower limb deep tendon reflexes				
Hyporeflexia	22 (71%)	0	25 (96%)	47 (67%)
Normoreflexia	9 (29%)	7 (54%)	1 (4%)	17 (24%)
Hyperreflexia	0	6 (46%)	0	6 (9%)
Babinski sign	0	2 (15%)	4 (15%)	6 (9%)
Laboratory findings				
Vitamin B12 (pmol/L), median (range)	151.0 (60–530)	167 (124–734)	168.5 (91–479)	159 (60–734)
Normal (>250 pmol/L)	3 (12%)	1 (8%)	7 (27%)	11 (16%)
Intermediate (160–250 pmol/L)	8 (26%)	7 (54%)	7 (27%)	22 (31%)
Low (<160 pmol/L)	20 (65%)	4 (31%)	10 (38%)	34 (49%)
Missing	0	1 (8%)	2 (8%)	3 (4%)
Methylmalonic acid (μmol/L)^b^, median (range)	2.23 (0.83–72.15)	3.58 (0.15–35.0)	2.91 (0.43–20.06)	2.66 (0.15–72.2)
EDx				
Only abnormal myography/performed	8/29 (27.5%)	0	2/23 (9%)	10/57 (17.5%)
Polyneuropathy/performed	18/29 (62%)	4/5 (80%)	20/23 (87%)	42/57 (74%)
MRI spine				
Myelopathy present/performed	4/17 (24%)	10/12 (83%)	16/23 (70%)	30/52 (58%)
Cervical	4 (100%)	9 (90%)	16 (100%)	29 (97%)
Cervical and thoracic	0	1 (10%)	0	1 (3%)
Involving ≤4 segments	1 (25)	1 (10%)	4 (25%)	6 (20%)
Involving ≥5 segments	3 (75%)	9 (90%)	12 (75%)	24 (80%)

Abbreviations: EDx, electrodiagnosis; LL, lower limbs; MRC‐SS, Medical Research Council sum score; MRI, magnetic resonance imaging; PNP, polyneuropathy; SCD, subacute combined degeneration; UL, upper limbs.

^a^ In all cases a thoracic sensory level (Th1−Th9) was reported at neurological examination.

^b^ Methylmalonic acid of 0.00–0.32 μmol/L was considered normal, >0.32 elevated.

### Laboratory investigations

The median VB12 level of all patients was 159 pmol/L (range 60–734). The majority (34 patients, 49%) had low VB12 levels, 22 patients (31%) intermediate, 11 patients (16%) normal and the VB12 level was missing in three (4%). The median VB12 level and MMA level were not statistically significantly different between groups. Ten of the 11 patients (91%) with a normal VB12 level had elevated MMA of which six had reported to have started self‐initiated oral VB12 supplementation before presentation at our hospital. One patient had both normal VB12 (734 pmol/L) and MMA (0.15 μmol/L) levels but started oral supplementation of VB12 1 month prior to the hospital visit.

### Magnetic resonance imaging features

Magnetic resonance imaging of the cervical spine was performed for 52 patients (74%) with abnormalities in 30 (58%). All showed T2‐weighted signal enhancement affecting the dorsal columns of the cervical spine including edema of the cervical spine in four (13%). The majority (21 patients, 70%) had a cervical myelopathy involving five or six segments. One patient had a cervical and thoracic myelopathy going as far as the tenth thoracic. In 4/17 patients (24%) with a clinically isolated PNP the MRI showed a myelopathy. In clinical signs of SCD (with or without PNP) MRI of the spine was normal in 9/35 patients (26%).

### Electrophysiological findings

**FIGURE 1:**
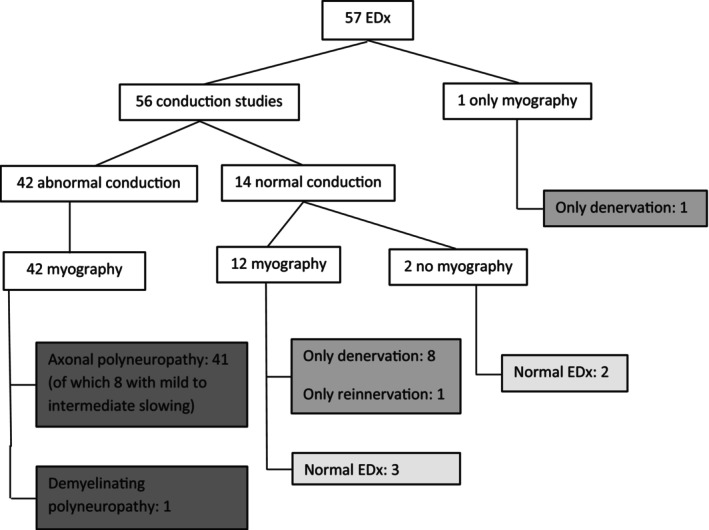
Electrodiagnostic results.

Electrodiagnosis was performed in 57/70 patients (81%). The median time between first presentation and EDx was 19 days (range 1–120 days) with a median of 71 days between first symptoms and EDx (range 2–577). Figure 1 gives an overview of the EDx results.

#### Motor NCS

Table 3 shows the motor NCS results. Motor NCSs were performed in 55 patients. The median amount of motor nerves tested per patient was 3 (range 0–3). In 22% (12/55 patients) motor NCSs were normal. The peroneal nerve showed a severely reduced distal compound muscle action potential (dCMAP) amplitude or was absent in 65% (36/55) and in 67% (36/54) for the tibial nerve. The median nerve was less often involved; the dCMAP amplitude was severely reduced in 26% (14/53) for the median nerve. All these patients also had severely reduced or absent dCMAP amplitudes in the peroneal and tibial nerves.

**TABLE 3:** Electrodiagnostic studies: motor nerve conduction studies.

	Nerve		
	Median (m. APB) (*n* = 53)	Peroneal (m. EDB) (*n* = 55)	Tibial (m. AH) (*n* = 54)
DML (ms)			
Median (range)	3.7 (3.0–8.0)	5.4 (3.3–11.1)	5.0 (3.4–10.3)
Normal	46 (87%)	29 (72.5%)	37 (82%)
101%–120% ULN	5 (9%)	8 (20%)	4 (9%)
120%–150% ULN	1 (2%)	2 (5%)	2 (4%)
>150% ULN	1 (2%)	1 (2.5%)	2 (4%)
dCMAP amplitude (mV)			
Median (range)	6.9 (1.8–14.2)	1.00 (0.0–10.0)	2.1 (0.0–16.2)
Normal	33 (62%)	16 (29%)	17 (31%)
80%–99% LLN	6 (11%)	3 (5.5%)	1 (2%)
<80% LLN	14 (26%)	21 (38%)	27 (50%)
Absent	0	15 (27%)	9 (17%)
MCV (m/s)			
Median (range)	53.4 (29.4–65.3)	43.0 (21.7–49.2)	40.2 (29.5–57.9)
Normal	37 (70%)	22 (69%)	25 (66%)
80%–99% LLN	14 (26%)	8 (25%)	11 (29%)
70%–79% LLN	1 (2%)	1 (3%)	1 (2.5%)
<70% LLN	1 (2%)	1 (3%)	1 (2.5%)
F wave (ms)			
Proportion performed	78%	20%	37%
Median minimal latency in ms (range)	28.4 (24.8–35.8)	52.4 (44.6–58.1)	58.5 (44.9–86.0)
Normal	32 (78%)	11 (100%)	11 (55%)
Latency >100%–120% ULN	8 (20%)	0	8 (40%)
Latency >120% ULN	1 (2%)	0	1 (5%)

Abbreviations: dCMAP, distal compound muscle action potential; DML, distal motor latency; LLN, lower level of normal; m. AH, muscle abductor hallucis; m. APB, muscle abductor pollicis brevis; m. EDB, muscle extensor digitorum brevis; MCV, motor nerve conduction velocity; ULN, upper level of normal.

Demyelinating features according to the EAN/PNS criteria (see Data S1) were only present in one patient. This patient had uniformly distributed, severe conduction slowing in all investigated nerves (prolonged distal motor and F wave latencies and reduced motor nerve conduction velocity in median, peroneal and tibial nerve), with a family history of PNP and hammertoes. Charcot−Marie−Tooth (CMT) type 1a was genetically confirmed (duplication in the *PMP22* gene). Mild to intermediate conduction slowing, not fulfilling the EAN/PNS demyelinating criteria, was present in 10 nerves in eight patients, in 80% of nerves accompanied by low dCMAP amplitude (<1 mV) and/or low temperature (<30°C).

#### Sensory NCS

Table 4 shows the sensory NCS results of 57 patients. The amount of sensory nerves tested per patient varied (median 3, range 0–4). In the legs sensory nerve action potential (SNAP) amplitudes were often reduced or absent (61% of the superficial peroneal nerves and 38% of the sural nerves). In 62% of sural nerves amplitudes were normal (reference value >4 μV), with amplitudes >10 μV in 39.6% (21/53). In 22.6% (12/53) sural amplitudes were between 4 and 10 μV. Normal SNAP amplitudes of the arm nerves were seen in the majority of patients (78% of the median and 83% of the ulnar nerves).

**TABLE 4:** Electrodiagnostic studies: sensory nerve conduction studies.

	Nerve			
	Median, *n* = 45	Ulnar, *n* = 18	Sural, *n* = 53	Peroneal superficial, *n* = 38
SNAP amplitude (mV)				
Median (range)	15.8 (0.0–71)	15.8 (0.0–31.7)	8.0 (0.0–42.6)	4.7 (0.0–29.7)
Normal	35 (78%)	15 (83%)	33 (62%)	15 (39%)
Decreased	9 (20%)	2 (11%)	12 (23%)	12 (32%)
Absent	1 (2%)	1 (6%)	8 (15%)	11 (29%)
SCV (m/s)				
Median (range)	47.65 (30.9–61.3)	48.9 (37.7–56.9)	37.4 (4.6–49.8)	37.7 (29.2–45.9)
Normal	30 (77%)	6 (75%)	10 (40%)	7 (41%)
Decreased	9 (23%)	2 (25%)	15 (60%)	10 (59%)

Abbreviations: SCV, sensory nerve conduction velocity; SNAP, sensory nerve action potential.

In 43% (24/57 patients) the sensory NCS was normal in arms and legs. In 39% (22/57 patients) abnormal sensory NCS was confined to the nerves of the lower extremities. In only one patient sensory NCS was abnormal with normal motor NCS and myography. This patient with a proven VB12 deficiency also reported drinking 6 alcohol units daily.

Hoffmann's reflex was absent in 47% (27 patients), normal in 39% (22 patients) and missing in 15% (eight patients).

#### Myography

Myography was performed in 96% (55 patients), with tibial anterior muscle tested in all patients (100%) and first dorsal interosseous muscle of the foot in 93% (51/55). All patients with abnormal motor conduction showed signs of denervation. No fasciculation potentials were observed. Signs of re‐innervation were seen in 16% (9/55 patients); all showed polyphasic motor unit potentials with or without prolonged duration. In 24% (13/55 patients) severely reduced recruitment of motor units was seen in the tibial anterior muscle, with increased firing rate on the remaining motor units. In 64% (9/14 patients) with normal sensorimotor conduction, myography showed signs of denervation (57%) and/or re‐innervation (7%).

#### Electrodiagnosis classification

In 91% (52/57 patients) EDx showed abnormalities (see Figure 1). In 74% (42/57) PNP criteria were fulfilled: 97.6% (41/42) were diagnosed with an axonal PNP and one patient with demyelinating subtype (CMT1a). In 20% (8/41) mild to intermediate slowing was present, not fulfilling demyelinating criteria.

In 38% (16/42) motor NCSs were abnormal with normal sensory NCSs. In 62% (26/42) both motor and sensory NCSs were abnormal. In 24% (10/42) dCMAP amplitude of the peroneal nerve and/or tibial nerve were (close to) absent (0%–10% of the lower limit of normal) with relatively spared sensory amplitudes of sural and/or superficial peroneal nerves (median amplitudes 81% of the lower limit of normal).

In 25% (14/56) both motor and sensory conduction was normal, not fulfilling the PNP criteria, even though 64% (9/14) showed abnormal myography (89% denervation, 11% re‐innervation). In one patient only myography was performed due to discomfort on NCS, showing signs of denervation in both leg muscles.

## DISCUSSION

A large retrospective cohort of 70 patients with N_2_O‐induced PNP and/or myelopathy is presented. This study accurately describes the clinical and diagnostic features including MRI of the spine, extensive EDx data with myography performed in the majority of patients.

Our cohort, in concordance with the literature, involved a young aged population with a male, non‐Caucasian predominance [5, 6, 14, 16, 17, 19, 20, 23, 30]. There was a wide range of duration and total N_2_O intake before presentation, which has been published previously [17, 20, 23, 31, 32]. Most patients presented with a delay of at least 1 month after onset of symptoms. Data on prognosis in the literature are often insufficient because either follow‐up is not conducted [25] or there is a high rate of loss to follow‐up [23, 24]. For example, Yu et al. reported 54% loss to follow‐up in their cohort study of 110 patients [23]. On average the prognosis is found to be good with percentages of recovery varying between 67% and 95% [12, 23, 24]. In our study, a large proportion of patients (81%) improved completely or with mild residual symptoms. This highlights the importance of education and VB12 supplementation [6] and prompt abstinence of N_2_O abuse [8, 11, 14, 17, 23, 24, 32].

Clinically, the majority of patients presented with sensory more than motor complaints, with legs more involved than arms and with walking difficulties [5, 6, 8, 14, 16, 20, 24, 31]. Micturition problems were reported in 20%, in patients with both proven PNP and/or SCD. It is hypothesized that micturition problems in patients with N_2_O abuse can be caused by PNP with autonomic involvement or due to spinal cord damage [16]. Patients were clinically diagnosed with isolated PNP in 44% (31 patients), isolated SCD in 19% (13 patients) and combined PNP and SCD in 37% (26 patients). However, our results showed that clinical discrimination between PNP and SCD is insufficient, because patients with clinically isolated SCD showed EDx abnormalities in 80% and in cases with clinically isolated PNP 24% of MRI revealed a myelopathy. This underlines the importance of further investigation by EDx and MRI of the (cervical) spine when neurological sequelae due to N_2_O are suspected.

In 16% (11/70) of patients VB12 levels were within the normal range (VB12 > 250 pmol/L), but in 91% (10/11) VB12 levels were accompanied by elevated MMA. A systematic review by Marsden et al. including N_2_O‐induced myeloneuropathy cases reported 243 VB12 values of which 52% were normal [8]. This can be explained by self‐initiated prophylactic VB12 supplementation, which might mask or insufficiently treat VB12 deficiency [8, 13, 33]. In our cohort 64% (7/11) of patients with normal VB12 levels reported using oral VB12 supplementation. This is likely to be underreported. Our study underlines the importance of MMA and/or homocysteine (HC) measurements, as VB12 levels might be normal in N_2_O abuse [1, 8, 14, 16, 33, 35].

On MRI the dorsal column of the cervical spine is known to be most prone for N_2_O damage and in a smaller proportion also the thoracic spine [6, 8, 22]. In our cohort MRI of the cervical spine showed a myelopathy in 58% in, on average, five to six segments which is also reported in the literature [24, 34]. Normal MRI was observed in 26% of cases with clinical signs of myelopathy. The latter might be explained by supplementation of VB12 prior to MRI [35].

Electrodiagnosis abnormalities were present in 91% of our patients (52/57) with or without clinical symptoms of PNP. All but one (CMT1a) classified as an axonal PNP [5, 12, 19]. Although 20% showed mild to intermediate conduction slowing, the EDx demyelinating criteria were not met. This mild conduction slowing might well be explained by axonal loss and/or low limb temperature (<30°C) in 80%. Pathology studies showed primarily axonal damage with chronic axonal damage with secondary segmental demyelinating in late stages at sural nerve biopsy in both VB12 PNP and specifically in N_2_O‐induced PNP [22, 31]. Pure demyelinating PNP in N_2_O abuse has been described in up to 30% of cases in the literature [9, 12, 19, 20, 23]. Despite this, in our cohort demyelinating PNP due to N_2_O abuse was not present. The discrepancy is likely to be explained by the use of different, less strict, demyelination EDx criteria [12, 19, 23] or these criteria were not specified [5, 6, 18, 20, 24].

Motor EDx abnormalities were more prominent than sensory abnormalities in 62% of patients with EDx‐proven PNP. Seventeen percent (9/52) of patients had normal sensorimotor conduction but abnormal myography which underlines the importance of myography in N_2_O‐induced PNP. Motor predominant NCS abnormalities have been described before in 38%–47% patients [5, 12, 17, 18, 19, 36]. Berling et al. reported a strong association between elevated HC plasma levels and severe motor axonal neuropathies [17]. This could not be reproduced in our study, because HC levels were lacking as only MMA levels were measured. The high prevalence of sensory symptoms in our cohort (97%) does not support this hypothesis. More pronounced motor axonal dysfunction and less prominent sensory changes were found in N_2_O‐abuse PNP compared to PNP by VB12 deficiency by other causes [36, 37, 38]. It was hypothesized that a direct toxic effect of N_2_O was responsible for this, and therefore this differed from damage by VB12 deficiency alone [36, 38]. Gao et al. found significantly higher serum VB12 levels in patients with N_2_O‐related SCD compared to SCD unrelated to N_2_O [34]. This hypothesis might explain normal VB12 levels in some patients. In our view, the combination of prominent sensory symptoms and selective motor EDx abnormalities might also indicate involvement of the nerve roots in N_2_O‐abuse PNP.

As reference values for sensory NCSs are known to be quite variable, this could also partly contribute to the deviation between motor and sensory NCSs. Therefore, normal sensory amplitudes according to strict reference values might be an underestimation of abnormal sensory NCSs. This was also suggested by Lee et al. [36]. Further research is warranted to clarify the more prominent motor NCS abnormalities found in N_2_O‐abuse patients. Follow‐up EDx studies might help to partially overcome this problem of sensory reference values, as ongoing sensory axonal degeneration will give rise to further decrement of sensory amplitudes and eventually become abnormal.

Our study has limitations. This is a retrospective study with clinical and diagnostic data not performed in a standardized manner. Despite this, the majority of patients underwent both EDx and MRI of the spine. It is uncertain whether the reported amount and duration of N_2_O use is correct and therefore no correlation could be made with the severity of symptoms and EDx changes. Unfortunately, it is well known that in the N_2_O‐abuse population there is a large proportion lost to follow‐up, also seen in our cohort (40%) [14, 23, 24]. Therefore, data about ongoing N_2_O abuse were insufficient. N_2_O is increasingly recognized as an addictive substance and this might explain the loss to follow‐up [39].

## CONCLUSION

Neurological sequelae due to N_2_O predominate in a young, male and non‐Caucasian population. VB12 levels can be normal, for example due to self‐initiated supplementation; therefore MMA and/or HC should be tested. The clinical distinction between PNP and SCD is often hampered by subclinical involvement of the peripheral nerves or dorsal columns, apparent from EDx and MRI studies. N_2_O abuse causes an axonal PNP, with more prominent motor than sensory abnormalities and in some patients abnormalities on myography with normal sensorimotor NCS studies. A demyelinating PNP features a red flag and should promptly point towards other causes.

## AUTHOR CONTRIBUTIONS

**L. T. Hassing:** Conceptualization; investigation; writing – original draft; methodology; writing – review and editing; formal analysis. **F. Y. Jiang:** Investigation. **R. Zutt:** Writing – review and editing; methodology; supervision. **S. Arends:** Writing – original draft; writing – review and editing; supervision.

## Supporting information


Data S1.

## Data Availability

The data that support the findings of this study are available from the corresponding author upon reasonable request.
