# Supplementary material for: Nitrous‐oxide‐induced polyneuropathy and subacute combined degeneration of the spine: clinical and diagnostic characteristics in 70 patients, with focus on electrodiagnostic studies
Source: Eur J Neurol. 2023 Sep 27;31(1):e16076. doi: 10.1111/ene.16076 (PMC11235686; doi:10.1111/ene.16076)
Supplement: Supplementary file 1 — Data S1. [file ENE-31-e16076-s001.docx]

**Supplementary data**

1. **EFNS/PNS CIDP electrodiagnostic criteria**

Motor nerve conduction criteria

At least one of the following:

(a) Motor distal latency prolongation ≥50% above ULN in two nerves (excluding median neuropathy at the wrist from carpal tunnel syndrome), or

(b) Reduction of motor conduction velocity ≥30% below LLN in two nerves, or

(c) Prolongation of F-wave latency ≥20% above ULN in two nerves (≥50% if amplitude of distal negative peak CMAP <80% of LLN), or

(d) Absence of F-waves in two nerves (if these nerves have distal negative peak CMAP amplitudes ≥20% of LLN) + ≥1 other demyelinating parametera in ≥1 other nerve, or

(e) Motor conduction block: ≥30% reduction of the proximal relative to distal negative peak CMAP amplitude, excluding the tibial nerve, and distal negative peak CMAP amplitude ≥20% of LLN in two nerves; or in one nerve + ≥ 1 other demyelinating parametera except absence of F- waves in ≥1 other nerve, or

(f) Abnormal temporal dispersion: >30% duration increase between the proximal and distal negative peak CMAP (at least 100% in the tibial nerve) in ≥2 nerves, or

(g) Distal CMAP duration (interval between onset of the first negative peak and return to baseline of the last negative peak) prolongation in ≥1 nerves+ ≥1 other demyelinating parametera in ≥1 other nerve

• (LFF 2 Hz) median > 8.4 ms, ulnar > 9.6 ms, peroneal > 8.8 ms, tibial > 9.2 ms

• (LFF 5 Hz) median > 8.0 ms, ulnar > 8.6 ms, peroneal > 8.5 ms, tibial > 8.3 ms

• (LFF 10 Hz) median > 7.8 ms, ulnar > 8.5 ms, peroneal > 8.3 ms, tibial > 8.2 ms

• (LFF 20 Hz) median > 7.4 ms, ulnar > 7.8 ms, peroneal > 8.1 ms, tibial > 8.0 ms
